# Proportional Fair Trajectory Design and Resource Allocation for UAV-Assisted SWIPT System

**DOI:** 10.3390/s22239359

**Published:** 2022-12-01

**Authors:** Kanghyun Heo, Kisong Lee

**Affiliations:** Department of Information and Communication Engineering, Dongguk University, Seoul 04620, Republic of Korea

**Keywords:** proportional fairness, energy harvesting, trajectory, resource allocation, optimization

## Abstract

In this study, we investigate the proportional fair trajectory design and resource allocation for an unmanned-aerial-vehicle (UAV)-assisted simultaneous wireless information and power transfer (SWIPT) system, where multiple ground nodes (GNs) receive information and harvest energy from the signal transmitted by the UAV using a power-splitting (PS) policy. With this system, we aim to maximize the sum of the logarithmic average spectral efficiency (SE) of the GNs while guaranteeing the average harvested energy requirement to improve the average SE and user fairness simultaneously. To deal with the nonconvexity of the optimization problem, we adopt the quadratic transform and first-order Taylor expansion, proposing an iterative algorithm to find the optimal trajectory and transmit the power of the UAV and the PS ratio of the GNs. Through simulations, we confirm that the proposed scheme achieves a higher average SE compared with the conventional baseline schemes and ensures a level of user fairness similar to that of the state-of-the-art baseline scheme.

## 1. Introduction

In recent years, unmanned aerial vehicles (UAVs) have emerged as a new architecture for next-generation wireless networks [1]. Compared to existing networks, in which a base station in a fixed location serves users, a UAV-assisted communication system provides many advantages, including low cost, high mobility, and good channel conditions owing to line-of-sight (LoS) transmission. Therefore, UAVs have been extensively deployed in various scenarios, such as mobile relays [2], emergency networks [3,4], and military applications [5].

In this context, several studies have been undertaken on the trajectory and resource allocation of UAVs [6,7,8]. In particular, the UAV trajectory was designed to minimize the mission completion time while ensuring a targeting file recovery probability [6], and an energy-efficient UAV trajectory was proposed with a propulsion energy consumption model for fixed-wing UAVs [7]. Moreover, an aerial cooperative jamming scheme including the design of trajectory and power control was proposed to maximize the average secrecy rate [8].

Because a UAV is highly mobile and can change its location freely over time, it can effectively shorten the distance to the ground node (GN) and increase the efficiency of wireless power transfer (WPT). Accordingly, UAV-enabled WPT and wireless information transfer have been simultaneously investigated [9,10,11,12,13,14]. In [9], the problem of maximizing the sum of energy collected by energy receivers was solved by optimizing the trajectory of the UAV. Further, in [10,11], the UAV strategies were optimized for a UAV-enabled wireless powered communication network, where each GN harvests energy from the signal transmitted by the UAV in the downlink and uses this harvested energy to send its information to the UAV in the uplink. In some works [12,13,14], a UAV-assisted simultaneous wireless information and power transfer (SWIPT) system has been considered, in which GNs receive information and harvest energy simultaneously from the signal sent by the UAV. In particular, the joint optimization of trajectory and transmit power was studied in [12] to maximize the average secrecy rate under the existence of separate information and energy receivers. Moreover, the resource allocation problems were considered to maximize the minimum average harvested energy [13] and the minimum average rate [14], respectively. Some recent works have proposed novel techniques based on artificial intelligence in predicting engineering complex design problems [15,16]. Although the UAV-assisted SWIPT system has been studied [13,14], UAV strategies have been devised by considering only max–min problems.

Accordingly, to present a more efficient UAV design strategy, we investigate the effect of the proportional fair trajectory and resource allocation on the UAV-assisted SWIPT system with a power-splitting (PS) policy. Compared to existing works on the UAV-assisted SWIPT system [12,13,14], the main contributions of our study are as follows:

- Considering that the improvements of the average spectral efficiency (SE), as well as user fairness are important for providing reliable quality-of-service to GNs, we formulate a problem to maximize the sum of the logarithmic average SE of the GNs while guaranteeing the average harvested energy requirement.
- To solve the formulated nonconvex problem, we convert the original problem into a tractable convex form using the quadratic transform and first-order Taylor expansion. In addition, we propose an iterative algorithm that finds the optimal trajectory and transmit power of the UAV, as well as the PS ratio of the GNs.
- Simulation results verify that the proposed scheme outperforms the state-of-the-art max–min scheme in terms of the average SE and provides a similar level of user fairness. This implies that the design of the UAV strategy in a proportional fair manner is effective in supporting fair and high data rates for GNs.

The remainder of this paper is organized as follows. In Section 2, we present a proportional fair resource allocation problem along with the model of the UAV-assisted SWIPT system. In Section 3, we propose an iterative algorithm for optimizing the trajectory design and resource allocation. In Section 4, we evaluate the performance of the proposed scheme through simulations. Finally, we provide the conclusions in Section 5.

## 2. System Model and Problem Statement

**Figure 1:**
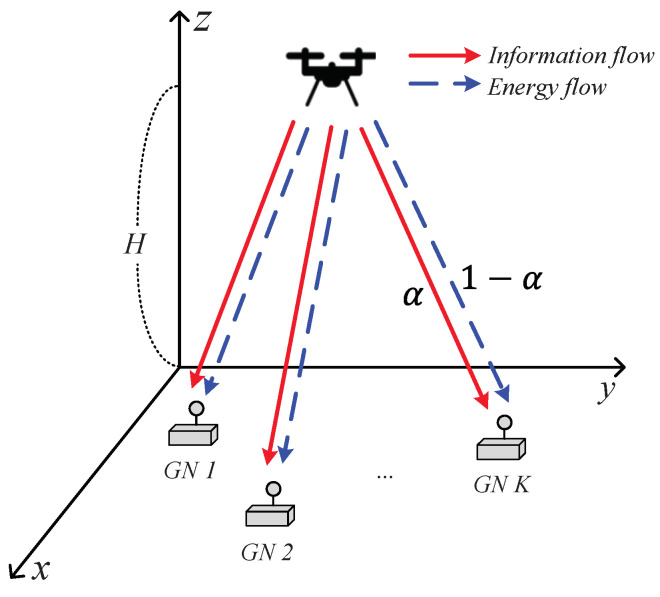
Model of a UAV-assisted SWIPT system.

As shown in Figure 1, we consider a UAV-assisted SWIPT system, in which a single UAV transmits information and power through the same signal to *K* GNs. Let *T* denote the UAV flight period, which is discretized into *N* equal-length time slots, $\delta =\frac{T}{N}$, and assume the location of the UAV to be unchanged within each time slot. We also denote *H* and *V* as the fixed altitude and maximum flying speed of the UAV; then, the maximum flying distance becomes L=Vδ in each time slot. The fixed horizontal coordinate of the GN *k* is given by $w_{k}={\left[x_{k},y_{k}\right]}^{T}\in R^{2\times 1},\;k\in K=\left\{1,2,\cdots ,K\right\}$, and the horizontal coordinate of the UAV at each time slot is expressed as $q\left[n\right]={\left[x\left[n\right],y\left[n\right]\right]}^{T}\in R^{2\times 1},\;n\in N$. Moreover, the UAV must periodically return to its starting position after one period to support the GNs. Then, the mobility constraints of the UAV are given by

(1) $$\begin{array}{cc} ∥q[n+1]-q[n]∥ & \leq L,\;\;\forall n\in N\setminus \{N\}, \end{array}$$

(2) $$\begin{array}{cc} q[1] & =q[N]. \end{array}$$

Given that the air-to-ground wireless channels are dominated by LoS links, the free-space path-loss model is adopted [9,10,11,12,13,14], where the Doppler effect caused by the mobility of the UAV is assumed to be completely compensated at the GNs. Then, the channel gain between the UAV and GN *k* at time slot *n* is expressed as

(3) $$\begin{array}{c} h_{k}\left[n\right]=\frac{\beta_{0}}{d_{k}^{2}\left[n\right]}=\frac{\beta_{0}}{∥q\left[n\right]-w_{k}{∥}^{2}+H^{2}},\;\;\forall k,\;n, \end{array}$$

where $\beta_{0}$ indicates the channel power gain at the reference distance of 1 m and $d_{k}\left[n\right]$ is the physical distance between the UAV and GN *k* at time slot *n*.

Let p[n] denote the transmit power of the UAV in time slot *n*; the average and peak power constraints should be satisfied, which are formulated as follows:

(4) $$\begin{array}{cc} \frac{1}{N}\sum_{n\in N}p\left[n\right] & \leq P_{\mathrm{avg}}, \end{array}$$

(5) $$\begin{array}{cc} 0\leq p[n] & \leq P_{\mathrm{peak}},\;\;\forall n, \end{array}$$

where $P_{\mathrm{avg}}$ and $P_{\mathrm{peak}}$ are the average and peak power budgets for the UAV, respectively.

For the functionality of the SWIPT, we consider a PS policy at the GNs, in which the portion $\alpha_{k}\left[n\right]$ of received RF signals at GN *k* in time slot *n* is used for harvesting energy and the remaining portion $1-\alpha_{k}\left[n\right]$ is used for receiving information. Then, the constraint for the PS ratio is given by

(6) $$\begin{array}{c} 0\leq \alpha_{k}\left[n\right]\leq 1,\;\;\forall k,\;n. \end{array}$$

From the formula of the Shannon capacity [17], the achievable SE from the UAV to GN *k* in time slot *n* is expressed as

(7) $$\begin{array}{ll} R_{k}\left[n\right] & =\log_{2}\left(1+\frac{\left(1-\alpha_{k}\left[n\right]\right)h_{k}\left[n\right]p\left[n\right]}{\left(1-\alpha_{k}\left[n\right]\right)\sigma_{A}^{2}+\sigma^{2}}\right)\\ & =\log_{2}\left(1+\frac{\beta_{0}\left(1-\alpha_{k}\left[n\right]\right)p\left[n\right]}{\left(\left(1-\alpha_{k}\left[n\right]\right)\sigma_{A}^{2}+\sigma^{2}\right)(∥q\left[n\right]-w_{k}{∥}^{2}+H^{2})}\right), \end{array}$$

where $\sigma^{2}$ and $\sigma_{A}^{2}$ are the powers of the baseband noise and antenna noise, respectively. The average SE from the UAV to GN *k* is also represented by

(8) $$\begin{array}{cc} {\bar{R}}_{k} & =\frac{1}{N}\sum_{n\in N}R_{k}\left[n\right],\;\;\forall k. \end{array}$$

Further, the harvested energy of GN *k* in time slot *n* is formulated as

(9) $$\begin{array}{ll} E_{k}\left[n\right] & =\delta \eta_{k}\alpha_{k}\left[n\right]h_{k}\left[n\right]p\left[n\right]\\ & =\frac{\gamma_{k}\alpha_{k}\left[n\right]p\left[n\right]}{∥q\left[n\right]-w_{k}{∥}^{2}+H^{2}}, \end{array}$$

where $\eta_{k}$ is the energy conversion efficiency of GN *k* and $\gamma_{k}=\delta \beta_{0}\eta_{k}$. The average harvested energy of GN *k* is also obtained as

(10) $$\begin{array}{cc} {\bar{E}}_{k} & =\frac{1}{N}\sum_{n\in N}E_{k}\left[n\right]. \end{array}$$

For the subsequent operation of GN *k*, we consider the average harvested energy requirement for each GN as follows:

(11) $$\begin{array}{c} {\bar{E}}_{k}\geq E_{\min},\;\;\forall k, \end{array}$$

where $E_{\min}$ is the minimum required harvested energy.

In this study, we consider proportional fair resource allocation to simultaneously increase the average SE of GNs and user fairness [18]. Given that resources are allocated to GNs in a way that increases the SE of the GNs fairly due to the property of the logarithmic function, we aim to maximize the sum of the logarithmic average SE of the GNs while guaranteeing the average harvested energy requirement for each GN by optimizing the trajectory Q≜{q[n],∀n} and the transmit power P≜{p[n],∀n} of the UAV jointly with the PS ratio of the GNs $A≜\{\alpha_{k}\left[n\right],\;\forall k,\;n\}$ as follows:

(12) $$\begin{array}{ccc} \left(P0\right): & \max_{Q,\;P,\;A} & \sum_{k\in K}\ln\left({\bar{R}}_{k}\right)\\ & s.t. & (1)-(6),\;\mathrm{and}\;(11). \end{array}$$

## 3. Proposed Algorithm

The optimization problem (P0) in (12) is a nonconvex problem because the objective function and the constraint (11) are not jointly concave with respect to Q, P, and A. Therefore, we solve the problem for each optimization variable by fixing the remaining variables.

### 3.1. Transmit Power Optimization

For fixed A and Q, the optimization problem can be reformulated for a single optimization variable P as

(13) $$\begin{array}{ccc} \left(P1\right): & \max_{P} & \sum_{k\in K}\ln\left({\bar{R}}_{k}\right)\\ & s.t. & (4),\;(5),\;\mathrm{and}\;(11). \end{array}$$

The problem (P1) is concave with respect to P and can, therefore, be effectively solved by existing convex solvers, e.g., CVX [19].

### 3.2. Power-Splitting Ratio Optimization

To efficiently find the PS ratio and trajectory, we first convert the objective of (P0) into a tractable form using the quadratic transform, which transforms the sum-of-ratios functions into the more tractable quadratic functions [20].

Through the quadratic transform with introducing the auxiliary variable $x_{k}\left[n\right]$, $R_{k}\left[n\right]$ can be translated into

(14) $$\begin{array}{cc} f_{k}\left[n\right] & =\log_{2}(1+2x_{k}\left[n\right]\sqrt{\beta_{0}\left(1-\alpha_{k}\left[n\right]\right)p\left[n\right]}-x_{k}^{2}\left[n\right]\left(\left(1-\alpha_{k}\left[n\right]\right)\sigma_{A}^{2}+\sigma^{2}\right)(∥q\left[n\right]-w_{k}{∥}^{2}+H^{2})). \end{array}$$

We note that $f_{k}\left[n\right]$ is equivalent to $R_{k}\left[n\right]$ because this transformation satisfies conditions C1–C4 of Theorem 1 in [20]. It is also more tractable to consider $f_{k}\left[n\right]$ because it is concave with respect to each optimization variable, $\alpha_{k}\left[n\right]$ and q[n]. Moreover, when p[n], $\alpha_{k}\left[n\right]$, and q[n] are fixed, $f_{k}\left[n\right]$ is also concave with respect to $x_{k}\left[n\right]$ because it is the logarithm of the negative quadratic function of $x_{k}\left[n\right]$ and the logarithm is a non-decreasing function. Therefore, we can find the optimal value of $x_{k}\left[n\right]$ from $\frac{\partial f_{k}\left[n\right]}{\partial x_{k}\left[n\right]}=0$ as follows:

(15) $$\begin{array}{cc} x_{k}^{*}\left[n\right] & =\frac{\sqrt{\beta_{0}\left(1-\alpha_{k}\left[n\right]\right)p\left[n\right]}}{\left(\left(1-\alpha_{k}\left[n\right]\right)\sigma_{A}^{2}+\sigma^{2}\right)(∥q\left[n\right]-w_{k}{∥}^{2}+H^{2})} \end{array}$$

For fixed P and Q, the optimization problem can be reformulated for a single optimization variable A as

(16) $$\begin{array}{ccc} (P2): & \max_{A,\;X} & \sum_{k\in K}\ln\left(\frac{1}{N}\sum_{n\in N}f_{k}\left[n\right]\right)\\ & s.t. & (6)\;\mathrm{and}\;(11), \end{array}$$

where $X≜\{x_{k}\left[n\right],\;\forall k,\;n\}$. The problem (P2) is concave with respect to each A and X. Therefore, we can iteratively optimize the primal variable A and the auxiliary variable X until both variables converge to stationary points. In particular, for a fixed A, X can be efficiently calculated in a closed form using (15), while for a fixed X, A can be solved by CVX.

### 3.3. Trajectory Optimization

Similar to solving A, the nonconvexity of the objective function with respect to Q in the problem (P0) can be dealt with using the quadratic transform of (14). However, the problem is still nonconvex with respect to Q even when the other variables are fixed because the constraint (11) is not a convex set. To address the constraint (11), the successive convex optimization technique can be applied, in which the original function is approximated by a more tractable function at a given local point in each iteration. Given that a convex function is lower-bounded by its first-order Taylor expansion at any point, we can derive the concave lower bound of $E_{k}\left[n\right]$ as follows:

(17) $$\begin{array}{cc} E_{k}\left[n\right] & \geq -\frac{\gamma_{k}\alpha_{k}\left[n\right]p\left[n\right]}{{\left(∥q^{\left(m\right)}\left[n\right]-w_{k}{∥}^{2}+H^{2}\right)}^{2}}\left(∥q\left[n\right]-w_{k}{∥}^{2}-{∥q^{\left(m\right)}\left[n\right]-w_{k}∥}^{2}\;\right)+\frac{\gamma_{k}\alpha_{k}\left[n\right]p\left[n\right]}{∥q^{\left(m\right)}\left[n\right]-w_{k}{∥}^{2}+H^{2}}\\ & ≜E_{k}^{\mathrm{LB}}\left[n\right], \end{array}$$

where $q^{\left(m\right)}\left[n\right]$ is the given trajectory of the UAV in the *m*-th iteration.

Therefore, for fixed P and A, the following convex optimization problem can be built for a single optimization variable Q as

(18) $$\begin{array}{ccc} (P3): & \max_{Q,\;X} & \sum_{k\in K}\ln\left(\frac{1}{N}\sum_{n\in N}f_{k}\left[n\right]\right)\\ & s.t. & \frac{1}{N}\sum_{n\in N}E_{k}^{\mathrm{LB}}\left[n\right]\geq E_{\min},\;\;\forall k\\ & & (1)\;\mathrm{and}\;(2). \end{array}$$

Here, Q and X can be updated iteratively similar to (P2), and then, the problem (P3) can also be effectively solved by convex solvers. Because the feasible region of (P3) is a subset of that of the original problem (P0), we can find a lower bound solution for (P0) from (P3).

In summary, we build three subproblems that are convex with respect to each optimization variable and find the optimal variable for each subproblem iteratively until convergence to solve the original nonconvex problem (P0). The detailed procedure for the proposed algorithm is summarized in Algorithm 1. Specifically, we initialize the indicator for the number of iterations, e.g., m=0, and all control parameters including $P^{\left(m\right)}$, $A^{\left(m\right)}$, and $Q^{\left(m\right)}$. For determined control parameters, $\left\{P^{\left(m\right)},A^{\left(m\right)},Q^{\left(m\right)}\right\}$, we first calculate the average SEs of GNs, ${\bar{R}}_{k}^{\mathrm{old}}={\bar{R}}_{k}\left(P^{\left(m\right)},A^{\left(m\right)},Q^{\left(m\right)}\right)$, ∀k. We also find the optimal transmit power by solving (P1) and update it as $P^{\left(m+1\right)}$. Next, for the determined control parameters, $\left\{P^{\left(m+1\right)},A^{\left(m\right)},Q^{\left(m\right)}\right\}$, we update X using (15), find the optimal PS ratio by solving (P2), and update it as $A^{\left(m+1\right)}$. Because the value of X can be different as $A^{\left(m+1\right)}$ is updated, this procedure is repeated until both X and $A^{\left(m+1\right)}$ converge. Similarly, for the determined control parameters, $\left\{P^{\left(m+1\right)},A^{\left(m+1\right)},Q^{\left(m\right)}\right\}$, we update X using (15), find the optimal trajectory by solving (P3), and update it as $Q^{\left(m+1\right)}$. This procedure is also repeated until both X and $Q^{\left(m+1\right)}$ converge. Finally, the average SEs of the GNs are updated by the obtained control parameters, ${\bar{R}}_{k}^{\mathrm{new}}={\bar{R}}_{k}\left(P^{\left(m+1\right)},A^{\left(m+1\right)},Q^{\left(m+1\right)}\right)$, ∀k, and *m* is updated as m←m+1. The procedure described above is repeated until the objective function converges, such that $|\sum_{k\in K}\ln\left({\bar{R}}_{k}^{\mathrm{new}}\right)-\sum_{k\in K}\ln\left({\bar{R}}_{k}^{\mathrm{old}}\right)|<\epsilon$.

The objective function of the problem is non-decreasing over the iterations and bounded by a finite value; therefore, Algorithm 1 can be guaranteed to converge. We note that the number of iterations for the interior point method for the worst case is $O\;\left(\sqrt{n}\log\left(1/\epsilon \right)\right)$, where *n* is the number of optimization variables and ϵ>0 is the convergence threshold, while the number of computations in each iteration is $O\;\left(n^{3}\right)$[21,22]. Therefore, the computational complexity of Algorithm 1 is $O\;\left(M{\left(KN\right)}^{3.5}\log\left(1/\epsilon \right)\right)$, where *M* is the number of iterations for the outer loop from Line 2 to Line 9. This indicates that the proposed algorithm has polynomial complexity with respect to *K* and *N*.
**Algorithm 1** Proposed algorithm.Initialize $P^{\left(m\right)}$, $A^{\left(m\right)}$, $Q^{\left(m\right)}$, and m=0**repeat** ${\bar{R}}_{k}^{\mathrm{old}}={\bar{R}}_{k}\left(P^{\left(m\right)},A^{\left(m\right)},Q^{\left(m\right)}\right)$, ∀k Find $P^{\left(m+1\right)}$ by solving (P1) for given $\left\{P^{\left(m\right)},A^{\left(m\right)},Q^{\left(m\right)}\right\}$ Update X using (15), and find $A^{\left(m+1\right)}$ by solving (P2) iteratively until convergence for given   $\left\{P^{\left(m+1\right)},A^{\left(m\right)},Q^{\left(m\right)}\right\}$ Update X using (15), and find $Q^{\left(m+1\right)}$ by solving (P3) iteratively until convergence for given   $\left\{P^{\left(m+1\right)},A^{\left(m+1\right)},Q^{\left(m\right)}\right\}$ ${\bar{R}}_{k}^{\mathrm{new}}={\bar{R}}_{k}\left(P^{\left(m+1\right)},A^{\left(m+1\right)},Q^{\left(m+1\right)}\right)$, ∀k Update m←m+1**until** Convergence, $|\sum_{k\in K}\ln\left({\bar{R}}_{k}^{\mathrm{new}}\right)-\sum_{k\in K}\ln\left({\bar{R}}_{k}^{\mathrm{old}}\right)|<\epsilon$

## 4. Simulation Results and Discussion

For the performance evaluation, all GNs were distributed over an area of 300×300 m. Moreover, the simulation parameters are summarized in Table 1 [10,12,13,14], and the following four schemes were considered for the performance comparison:

- Proposed scheme: The transmit power and trajectory of the UAV and the PS ratio of the GNs are determined using Algorithm 1.
- Max–min scheme: The transmit power and trajectory of the UAV and the PS ratios of the GNs are determined to maximize the minimum average achievable SE among all GNs while guaranteeing the average harvested energy requirement [14].
- Circular scheme: The UAV has a circular trajectory with a radius of area/4 centered at the geometric mean of the GNs’ coordinates. The transmit power of the UAV and the PS ratio of the GNs are determined in the same way as in the proposed scheme.
- Hover-and-fly scheme: The UAV hovers sequentially at the coordinates of the GNs and flies in a straight line from one user to the other at a constant speed. The transmit power of the UAV and the PS ratio of the GNs are determined in the same way as in the proposed scheme.

**Figure 2:**
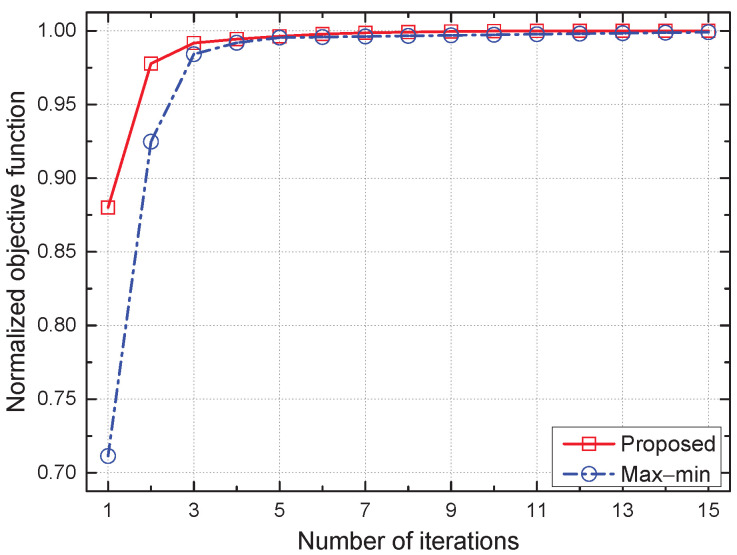
Convergence of the proposed scheme.

**Figure 3:**
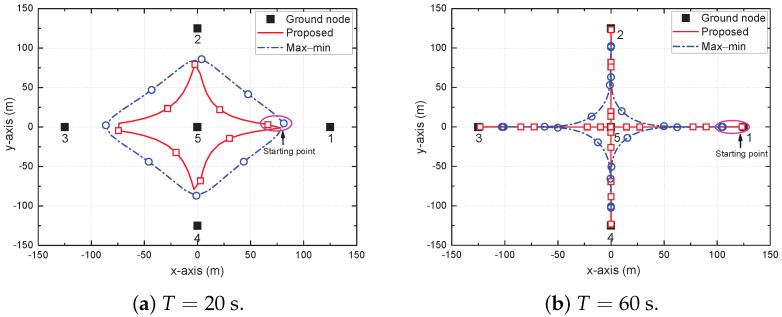
Trajectory of UAV for different *T*.

**Figure 4:**
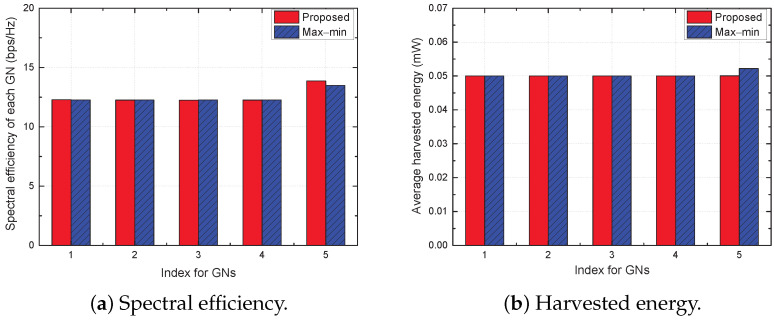
Spectral efficiency and harvested energy of each GN for T=60.

**Figure 5:**
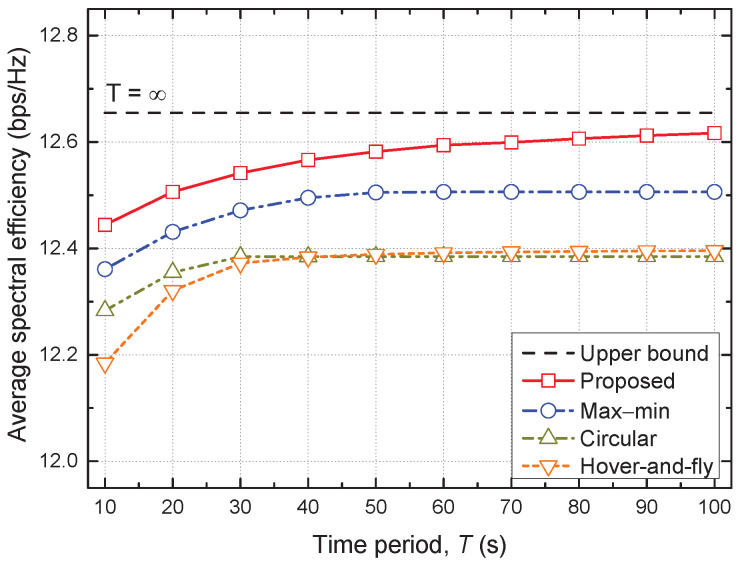
Average spectral efficiency vs. time period.

**Figure 6:**
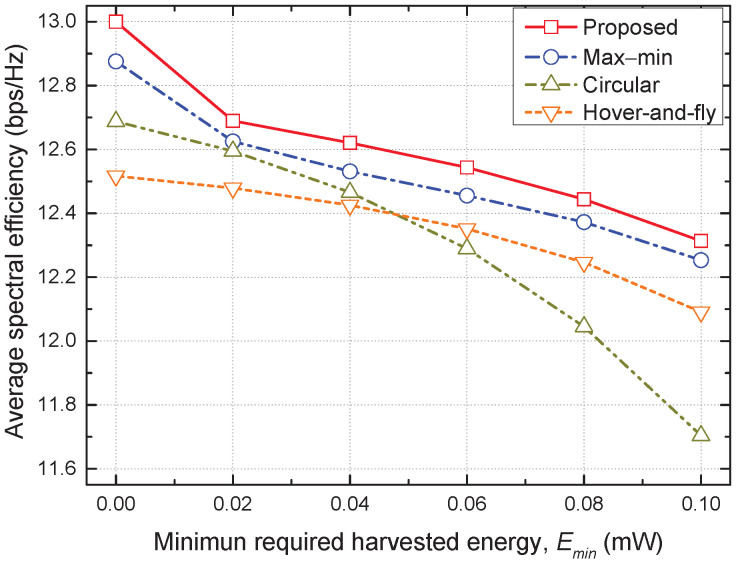
Average spectral efficiency vs. minimum required harvested energy.

In Figure 2, Figure 3 and Figure 4, we compare the distinguishing differences between the proposed and max–min schemes, where the latter is a state-of-the-art baseline scheme. Moreover, we compare the performance of all considered schemes in Figure 5 and Figure 6.

Figure 2 compares the convergence performance between the proposed and max–min schemes, where the objective function normalized to the convergence point is plotted on the y-axis because both schemes have different objectives, e.g., the sum of the logarithmic average SE of the GNs for the proposed scheme, while the minimum average SE among all GNs for the max–min scheme. Both schemes converge to the stationary point within 15 iterations; however, the proposed scheme has less variation and converges faster than the max–min scheme, which verifies the convergence stability of the proposed iterative algorithm.

Figure 3 shows the trajectory of the UAV for different *T*, e.g., T=20 s and T=60 s. The circular and square markers represent the positions of the UAV sampled every 2.5 s. When *T* is small, the UAV flies close to its maximum speed *V* to get as close to each GN as possible for building shorter LoS links within each limited time period. Moreover, as *T* increases, the UAV adjusts its trajectory to move closer to the GNs. Through this trajectory, the UAV can efficiently support all GNs. Compared to the max–min scheme, the trajectory of the proposed scheme is narrow and the UAV goes directly through the central GN. This is because the two schemes have different objectives. The max–min scheme should support all GNs as equally as possible to maximize the minimum average achievable SE; therefore, it should not go directly through the central GN, which is likely to receive the maximum service. However, to maximize the sum of the logarithmic average SE of the GNs, it is important to increase the SE of each GN, as well as to ensure the fairness of the GNs. Therefore, it allows the SE of the central GN to be greater than those of the other GNs to some extent so that the UAV visits all GNs directly.

Figure 4 shows the SE and harvested energy of each GN when T=60 for the proposed and max–min schemes. The proposed scheme guarantees the average harvested energy requirement tightly, but provides a higher SE for GN 5 compared with the other GNs. On the other hand, the max–min scheme supports GN 5 with a relatively low SE compared to the proposed scheme, but ensures higher harvested energy for GN 5. The reason for this aspect is that, as explained above, the objectives of the two schemes are different.

Figure 5 shows the average SE versus the time period *T* for all considered schemes. The average SE of all schemes increases with *T* and eventually saturates when *T* is large enough. In particular, the performance gap between the proposed scheme and its upper bound with T=∞ decreases as *T* increases. A larger *T* allows the UAV to fly for a longer time, making it easier for the UAV to get closer to the GNs for service. Accordingly, the average SE can be improved. However, the average SE of the circular and hover-and-fly schemes, which have a fixed trajectory, saturates earlier at a small *T*, and the performance improvement achievable by increasing *T* is also small. Furthermore, the proposed scheme outperforms the conventional baseline schemes, and the performance gap increases with *T*. In particular, the proposed scheme can serve the GNs with higher SE while providing a level of user fairness similar to that of the max–min scheme.

Figure 6 shows the average SE versus the minimum required harvested energy $E_{\min}$ for all considered schemes. As $E_{\min}$ increases, each GN must use a large portion of the received signal to harvest energy to ensure the requirement of the minimum harvested energy, resulting in a decrease in the achievable SE for all schemes. At a small $E_{\min}$, the hover-and-fly scheme shows the lowest SE, but reverses the performance of the circular scheme as $E_{\min}$ increases above 0.04. This implies that the hover-and-fly scheme provides more reliable service to the GNs than the circular scheme does for a large $E_{\min}$ because it directly visits all the GNs. Finally, the proposed scheme achieves the highest average SE over the entire range of $E_{\min}$, which validates the effectiveness of the proposed trajectory and resource allocation.

## 5. Conclusions

In this study, we considered a UAV-assisted SWIPT system with the PS policy and jointly optimized the trajectory and transmit power of the UAV and the PS ratio of the GNs to simultaneously improve the average SE of the GNs and the user fairness while ensuring the average harvested energy requirement for each GN. To tackle the nonconvexity of the optimization problem, we used the quadratic transform and first-order Taylor expansion, proposing an effective iterative algorithm. The simulation results showed that the proposed scheme outperforms the state-of-the-art max–min scheme in terms of the average SE and provides a similar level of user fairness. We expect our solution to open a new direction in the design of UAV strategies for a PS-based SWIPT system. For future work, our study can be extended to a multi-UAV SWIPT system where inter-UAV interference exists.

## Figures and Tables

**Table 1:** Parameter setup.

Parameter	Value
Flight period	T=60 s
Length of time slot	δ=0.5 s
Number of GNs	K=5
Flight altitude	H=50 m
Maximum flying speed	V=25 m/s
Average power budget	$P_{\mathrm{avg}}=40$ dBm
Peak power budget	$P_{\mathrm{peak}}=4P_{\mathrm{avg}}$
Minimum required harvested energy	$E_{\min}=0.05$ mW
Energy conversion efficiency	$\eta_{k}=0.5,\;\forall k$
Channel power gain at 1 m	$\beta_{0}=0$ dB
Antenna noise power	$\sigma_{A}^{2}=-70$ dBm
Baseband noise power	$\sigma^{2}=-40$ dBm

## Data Availability

Not applicable.
